# Type 1 and 3 collagen production by bone marrow mesenchymal stromal cells is suppressed *in vitro* after exposure to sepsis patient serum

**DOI:** 10.1186/2197-425X-3-S1-A639

**Published:** 2015-10-01

**Authors:** R Pernu, F Gäddnäs, J Risteli, T Ala-Kokko, P Lehenkari

**Affiliations:** department of anatomy and cell biology, University of Oulu, Oulu, Finland; ICU, Oulu University Hospital, Oulu, Finland; department of clinical chemistry, University of Oulu, Oulu, Finland

## Introduction

Severe sepsis is a systemic host response to invading pathogen with activated inflammation, coagulation and tissue remodelling cascades and consequent organ dysfunction. Despite modern intensive care, mortality in severe sepsis remains high. Mesenchymal stromal cells (MSCs) are known to have many anti-inflammatory features and contribute to the healing process. Many research groups have demonstrated good results using MSCs as an adjunctive treatment of sepsis in animal studies. Recently, a research group conducted a phase I clinical trial to test safety of MSCs on ARDS patients ([1]). To date knowledge of sepsis influence on MSCs is scarce.

## Objectives

Our aim was to study the capability of MSCs to produce extracellular matrix components, type 1 and 3 collagens, when exposed to serum of septic patients in an experimental *in vitro* sepsis model.

## Methods

Bone marrow MSCs (BM-MSC) were exposed to four different cell culture conditions: 1. Standard BM-MSC cell culture media (SCCM) with 20 % FBS, 2. SCCM with 20 % FBS added with 3 ng/ml TNF-alpha, 3. SCCM with 20 % human serum pooled from healthy volunteers (serum n = 8), 4. SCCM with 20 % human serum pooled from patients diagnosed with septic shock (serum n = 6). BM-MSCs were incubated for four days and the supernatant were collected and stored in -70° C until analysis. Production of type 1 and 3 collagens were determined by measuring the concentration of type 1 and 3 collagens N-terminal propeptide (PINP and PIIINP) from cell culture media. PINP concentration was determined with a chemiluminescence immunoassay (IDS iSYS, Immunodiagnostics Systems, Boldon, UK) and PIIINP concentration was determined with a radioimmunoassay (Orion Diagnostica, Espoo, Finland).

## Results

**Figure 1:**
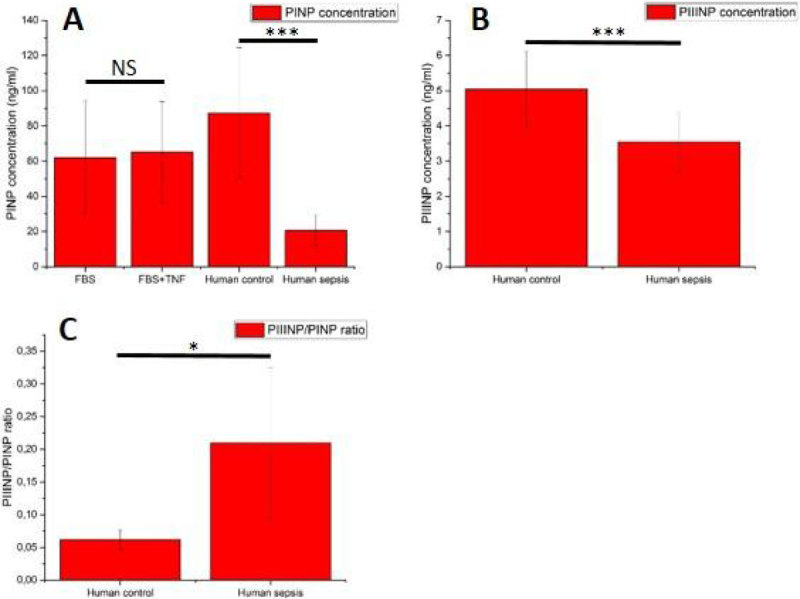
A. PINP concentration in FBS and human groups. B. PIIINP concentration in human groups. C. PIIINP/PINP ratio in human groups. *** = p < 0.001. * = p < 0.05.

In all BM-MSC lines, and repeated experiments (n = 6), production of both PINP and PIIINP were suppressed in the sepsis group (p < 0.001 both PINP and PIIINP). Addition of TNF-alpha, 3 ng/ml did not have effect on PINP levels in FBS groups. PIIINP/PINP ratio increased significantly in sepsis group compared to control group (p < 0.05) (Figure 1). Data is presented as mean ± SD.

## Conclusions

Serum from patients with severe sepsis suppresses the MSCs capability to produce collagen I and III *in vitro*. The PIIINP/PINP profile is also altered in favour of PIIINP over PINP. These results suggest that septic environment alters the extracellular matrix remodelling capability of MSCs, which in turn can alter tissue healing *in vivo*. This should be taken into consideration when using MSC therapy in severe sepsis.

## Grant Acknowledgment

Sigfrid Juselius Foundation, Oulu University Hospital's government allocated research funding.
